# Expression and Molecular Modification of Chitin Deacetylase from *Streptomyces bacillaris*

**DOI:** 10.3390/molecules28010113

**Published:** 2022-12-23

**Authors:** Lili Yin, Qi Wang, Jianan Sun, Xiangzhao Mao

**Affiliations:** 1 Qingdao Key Laboratory of Food Biotechnology, College of Food Science and Engineering, Ocean University of China, Qingdao 266404, China; 2 Key Laboratory of Biological Processing of Aquatic Products, China National Light Industry, Qingdao 266404, China; 3 Laboratory for Marine Drugs and Bioproducts of Qingdao National Laboratory for Marine Science and Technology, Qingdao 266237, China

**Keywords:** chitin deacetylase, molecular modification, chitin

## Abstract

Chitin deacetylase can be used in the green and efficient preparation of chitosan from chitin. Herein, a novel chitin deacetylase *Sb*CDA from *Streptomyces bacillaris* was heterologously expressed and comprehensively characterized. *Sb*DNA exhibits its highest deacetylation activity at 35 °C and pH 8.0. The enzyme activity is enhanced by Mn^2+^ and prominently inhibited by Zn^2+^, SDS, and EDTA. *Sb*CDA showed better deacetylation activity on colloidal chitin, (GlcNAc)_5_, and (GlcNAc)_6_ than other forms of the substrate. Molecular modification of *Sb*CDA was conducted based on sequence alignment and homology modeling. A mutant *Sb*CDA63G with higher activity and better temperature stability was obtained. The deacetylation activity of *Sb*CDA63G was increased by 133% compared with the original enzyme, and the optimal reaction temperature increased from 35 to 40 °C. The half-life of *Sb*CDA63G at 40 °C is 15 h, which was 5 h longer than that of the original enzyme. The improved characteristics of the chitin deacetylase *Sb*CDA63G make it a potential candidate to industrially produce chitosan from chitin.

## 1. Introduction

Chitin (chemically named β-(1,4)-2-acetylamino-2-deoxy-D-glucose) is the second most abundant natural polymer after cellulose and the most abundant renewable nitrogenous material on Earth [1,2]. It is widely found in crustaceans, insect exoskeletons, mollusks, and fungal cell walls [3]. The amount of chitin biosynthesized each year is approximately 100 billion tons, which can be regarded as an inexhaustible biological resource [4].

Chitosan (β-(1,4)-2-amino-2-deoxy-D-glucose) is the deacetylation product of chitin [5]. Due to the presence of amino groups, chitosan has better solubility than chitin [6]. Furthermore, it has good degradability and biocompatibility, and it also has a good hemostatic property, antibacterial property, moisturizing property, formability, and so on [7,8,9,10]. All of the properties mentioned above make chitosan widely used in food processing and preservation, wastewater treatment, medical material, agricultural planting, and many other fields [11,12,13,14].

In industry, chitosan is traditionally prepared by treating chitin with a concentrated alkali [15]. The quality of chitosan is mainly affected by the concentration of the alkali solution, pyrolysis time, and temperature during the reaction [16,17]. The alkali treatment method creates a large amount of environmental pollution, and it is furthermore difficult to control the molecular weight of the product. Consequently, the homogeneity of the produced chitosan is poor, and meeting requirements for the high-quality chitosan in biological and medical fields is difficult [18]. On the contrary, the enzymatic preparation of chitosan from chitin has attracted attention because of its green and controllable reaction process.

Chitin deacetylases (CDA) from the carbohydrate esterase 4 (CE4) family have attracted wide attention because of their ability to remove the acetyl group and produce chitosan or chitooligosaccharide using chitin or chitinosaccharides [19,20,21,22]. However, the studies and research progress regarding CDA are less advanced than those of chitosanase and chitinase. At present, a CDA that has high efficiency and good stability and is suitable for industrial application has not been discovered or developed [23,24,25,26,27,28,29,30,31,32,33]. There is an urgent need to excavate more CDAs and investigate their functional properties with the purpose of furthering the understanding of CDAs and developing CDAs for industrial applications.

In this study, a gene encoding chitin deacetylase was extracted from the genome of *Streptomyces bacillaris* and heterologously expressed in *Bacillus subtilis* WB800. The properties of chitin deacetylase were comprehensively studied, and the structure of chitin deacetylase was modified using single-point mutation and saturation mutagenesis. A modified enzyme with improved catalytic properties was obtained. These results provide a good reference for the exploration and molecular modification of new chitin deacetylases.

## 2. Results

### 2.1. Bioinformatics Analysis of SbCDA

**Figure 1:**
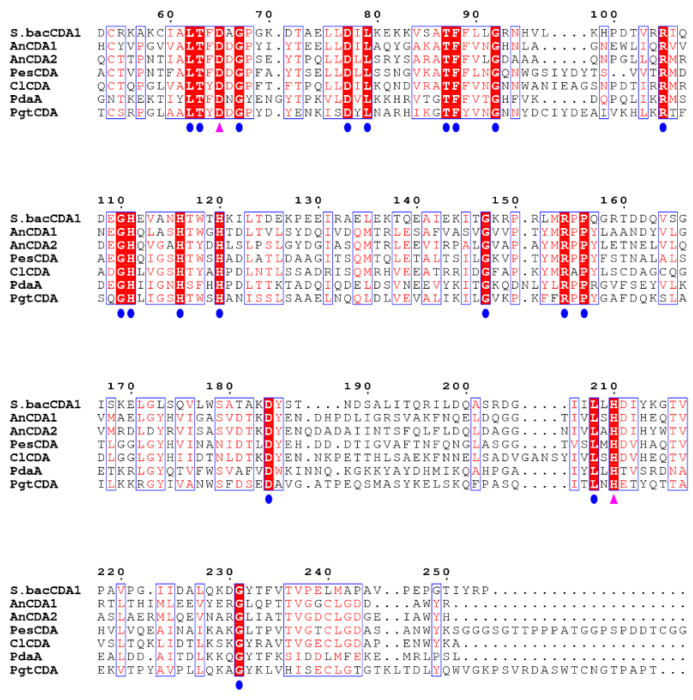
Protein sequence alignment of *Sb*CDA with PesCDA (Genbank: A0A1L3THR9) [34], PdaA (Genbank: AFQ56715) [25], ClCDA (Genbank: Q6DWK3) [35], AnCDA (Genbank: XP_659456) [30], PgtCDA (Genbank: XP_003323413) [36], and AnCDA2 (Genbank: ACF22101.1). Residues D and H playing catalytic roles are marked with purple triangles; the remaining 19 conserved sites are marked with blue dots.

The open reading frame (ORF) of *Sb*CDA (the amino acid sequences have been provided in Appendix B) consists of 867 nucleotides, encoding a protein of 289 amino acid residues. The molecular mass and isoelectric point of *Sb*CDA were predicted to be 31.37 kDa and 8.60, respectively. To analyze the key catalytic sites, we compared the protein sequence of *Sb*CDA with those of other chitin deacetylases (PesCDA (Genbank: A0A1L3THR9) [34], PdaA (Genbank: AFQ56715) [25], ClCDA (Genbank: Q6DWK3) [35], AnCDA (Genbank: XP_659456) [30], PgtCDA (Genbank: XP_003323413) [36], and AnCDA2 (Genbank: ACF22101.1)). There were 21 completely conserved amino acid sites among these CDAs (as shown by the red squares in Figure 1).

### 2.2. Expression and Purification of SbCDA

**Figure A1:**
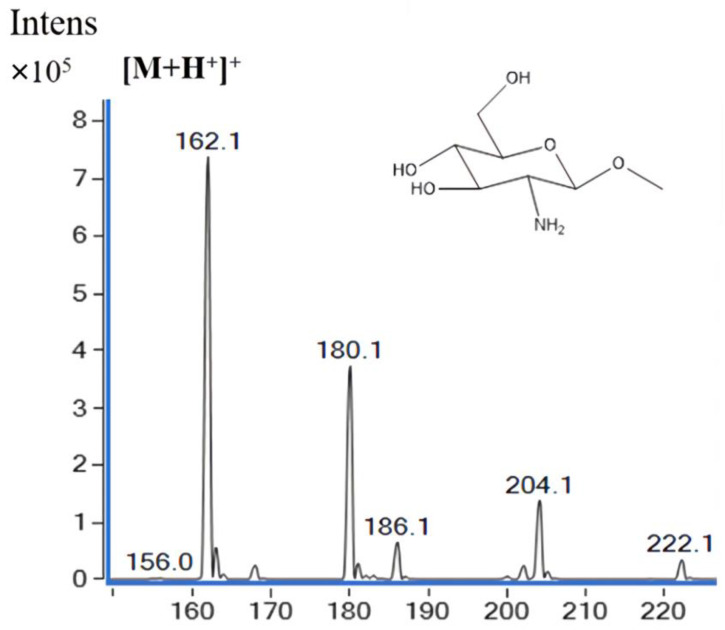
MS analysis of products of GlcNAc.

**Figure A2:**
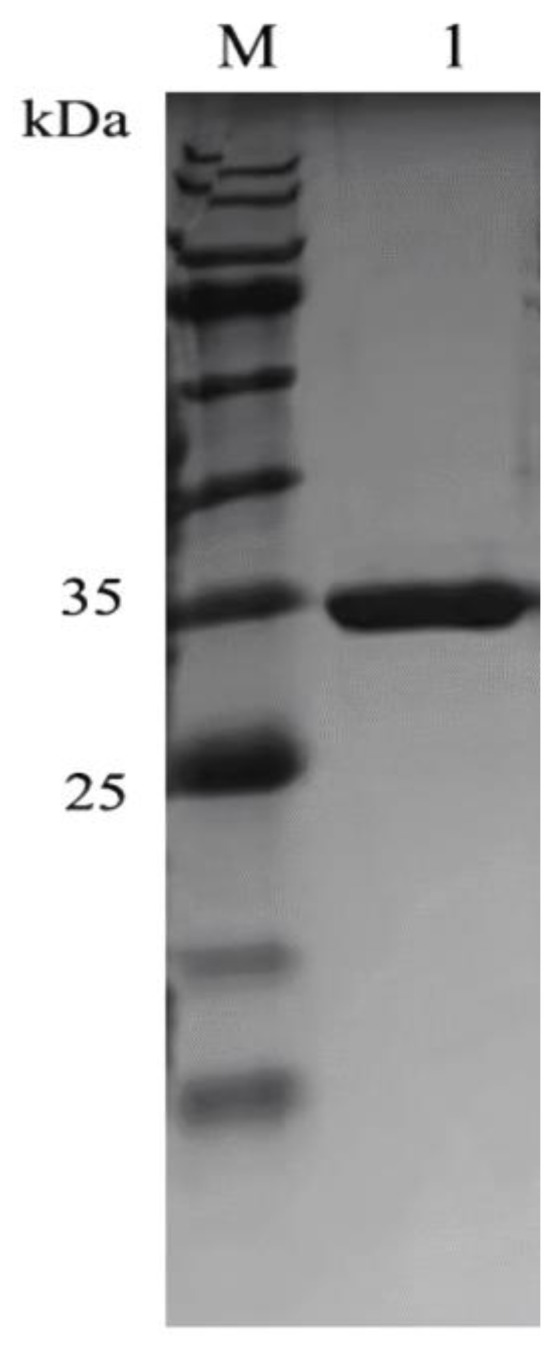
SDS-PAGE analysis of purified *Sb*CDA. M, molecular weight marker; 1, purified *Sb*CDA.

*Bacillus subtilis* is often used as the traditional fermentation host in industrial production owing to its safety, efficient protein secretion system, and short fermentation period [37]. *B. subtilis* WB800 is a commonly used expression host which is knocked out of eight extracellular protease genes for the purpose of avoiding the hydrolysis of the target protein [38]. The gene encoding the original *Sb*CDA was cloned and successfully expressed in *B. subtilis* WB800 with a C-terminal His tag, and the recombinant *Sb*CDA was purified using a Ni-NTA Superflow column. The protein concentrations of crude enzyme and purified *Sb*CDA were 2.2 mg/mL and 0.1 mg/mL, respectively. In addition, the specific enzyme activities of crude enzyme and purified *Sb*CDA under standard condition were 17.1 U/mg and 49.6 U/mg, respectively. The purification factor was 2.0. The product was identified using an Agilent 6460 LC/MS, and the results (Figure A1) confirmed the production of glucosamine (GlcN). SDS–PAGE analysis (Figure A2) showed that the obtained bands were consistent with the predicted results, with a molecular weight of approximately 32 kDa.

### 2.3. Characterization of SbCDA

#### 2.3.1. Effects of Temperature and pH on the Activity and Stability of SbCDA

**Figure 2:**
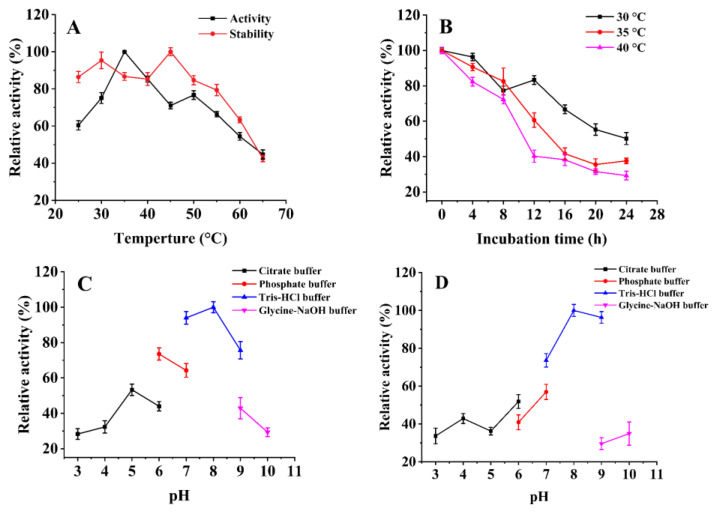
Biochemical characteristics of *Sb*CDA. (**A**) Effects of temperature on the activity of *Sb*CDA. Conditions for stability measurement: Tris-HCl buffer (10 mM, pH 7.0), 6 h. (**B**) Effects of temperature on the thermostability of *Sb*CDA. (**C**) Effects of pH on the activity of *Sb*CDA, citrate buffer (10 mM, pH 3.0–6.0), phosphate buffer (10 mM, pH 6.0–7.0), Tris-HCl buffer (10 mM, pH 7.0–9.0), and glycine-NaOH buffer (10 mM, pH 9.0–10.0). Reaction temperature: 40 °C. (**D**) Effects of pH on the stability in citrate buffer (10 mM, pH 3.0–6.0), phosphate buffer (10 mM, pH 6.0–7.0), Tris-HCl buffer (10 mM, pH 7.0–9.0), and glycine-NaOH buffer (10 mM, pH 9.0–10.0). Incubation conditions: 40 °C, 12 h. Using GlcNAc of 10 g/L as substrate throughout all measurements. All measurements were determined in triplicate, and the error bars indicate the standard deviations (*n* = 3).

The effects of temperature on *Sb*CDA were determined, and the results are shown in Figure 2A. The optimal temperature of *Sb*CDA was 35 °C under the reaction conditions of a pH 7.0 Tris-HCl buffer. The residual enzyme activity (Figure 2A) of *Sb*CDA was tested after incubation at different temperatures for 6 h. The results showed that the activity of *Sb*CDA could be maintained at about 85% at 35 °C and above 95% at 30 °C and 45 °C. Overall, the stability of *Sb*CDA was better below 50 °C, with the remaining enzyme activity being above 80% after 6 h. Residue activity of *Sb*CDA was measured every 4 h at 30, 35, and 40 °C; 24 h was the last time point to be analyzed. The results showed that the half-lives of *Sb*CDA (Figure 2B) at 30, 35, and 40 °C were 24 h, 14 h, and 10 h, respectively.

The effects of pH on the enzyme activity of *Sb*CDA were determined (Figure 2C). The optimum pH of *Sb*CDA was 8.0 in the Tris-HCl buffer solution. Due to the incubation temperature of 40 °C, under which the enzyme activity was maintained well, the incubation time was extended when measuring the stability of *Sb*CDA at different pH conditions. After incubation for 12 h, *Sb*CDA maintained more than 70% of its activity at pH values ranging from 7.0 to 9.0 (Figure 2D).

#### 2.3.2. Effects of Chemical Reagents on SbCDA Enzyme Activity

**Figure 3:**
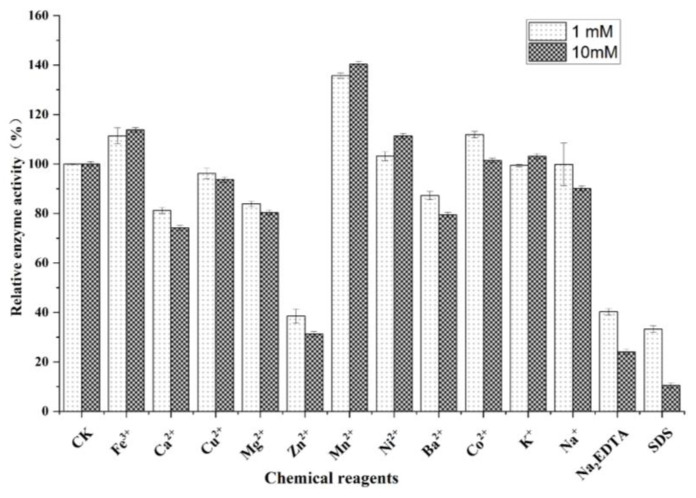
Effects of various chemical agents on enzyme activity of *Sb*CDA. CK represents the control group with nothing added. The incubation time of the enzyme with metal ions was 1 h at 37 °C. The reaction time for the enzyme with GlcNAc was 6 h in Tris-HCl buffer (10 mM, pH = 7.0) at 40 °C. All measurements were determined in triplicate, and the error bars indicate the standard deviations (*n* = 3).

To investigate the effects of different metal ions on *Sb*CDA and the tolerance of *Sb*CDA to protein-deformable agents and to confirm that chitin deacetylase is a metal ion-dependent enzyme, different metal ions (Fe^3+^, Ca^2+^, Cu^2+^, Mg^2+^, Zn^2+^, Mn^2+^, Ni^2+^, Ba^2+^, Co^2+^, K^+^, and Na^+^), SDS, and Na_2_EDTA were added into the reaction system, respectively. The results (Figure 3) showed that the activity of *Sb*CDA was strongly inhibited by Zn^2+^ and was mildly inhibited by Ca^2+^, Cu^2+^, Mg^2+^, Ba^2+^, and Na^2+^. The inhibition became stronger at high concentrations. Fe^3+^, Ni^2+^, and Co^2+^ slightly improved the activity of *Sb*CDA, while Mn^2+^ strongly improved the activity of *Sb*CDA. K^+^ inhibited *Sb*CDA activity at 1 mM but enhanced the activity at 10 mM. SDS and Na_2_EDTA also strongly inhibited the activity of *Sb*CDA.

### 2.4. Substrate Preference of SbCDA

**Figure 4:**
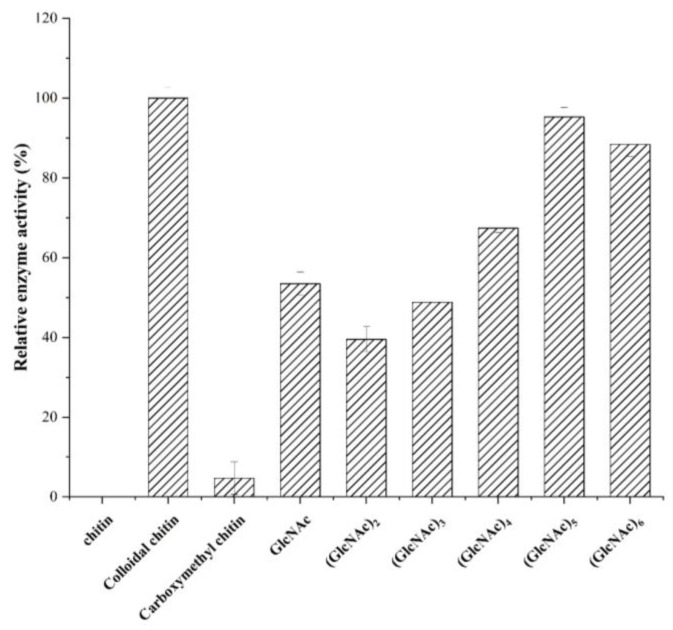
Determination of substrate preference of *Sb*CDA. Measured under standard conditions with 10 mg/mL of substrates. 100% activity was used for the best-performing substrate. All measurements were determined in triplicate, and the error bars indicate the standard deviations (*n* = 3).

By selecting chitin oligosaccharides with different degrees of polymerization and chitins with different solubilities as reaction substrates, the enzyme activity of *Sb*CDA with different substrates was determined. As shown in Figure 4, *Sb*CDA showed activity against colloidal chitin and soluble chitin oligosaccharides and had no effect on insoluble powder chitin. Compared with carboxymethyl chitin, GlcNAc, and (GlcNAc)_2–4_, *Sb*CDA clearly prefers colloidal chitin, (GlcNAc)_5_, and (GlcNAc)_6_.

### 2.5. Comparison of Enzyme Activity of Mutants and Analysis of Homologous CDAs

**Figure 5:**
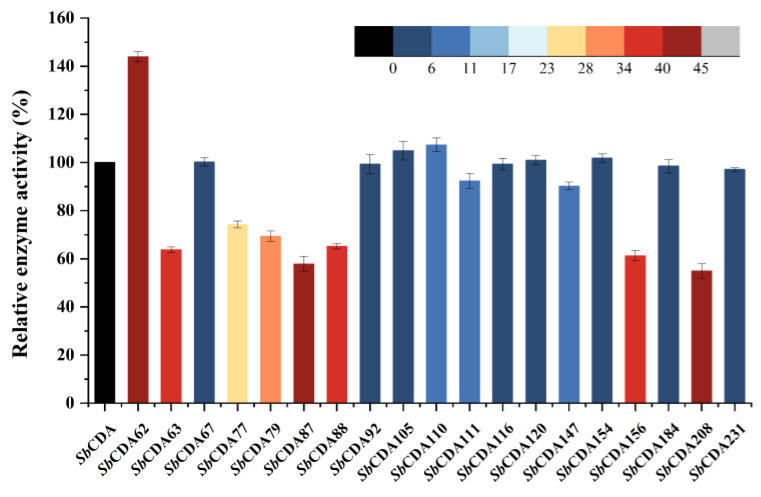
Relative enzyme activity after single-point mutation. Colors indicate the degree of change in enzyme activity (increase or decrease). The activity of *Sb*CDA was defined as 100%. The activity of mutants was obtained by comparison with *Sb*CDA. The redder the color, the more the enzyme activity was changed, and blue represents the opposite. Measurements were conducted under standard conditions using 10 mg/mL of GlcNAc. All measurements were determined in triplicate, and the error bars indicate the standard deviations (*n* = 3).

**Figure 6:**
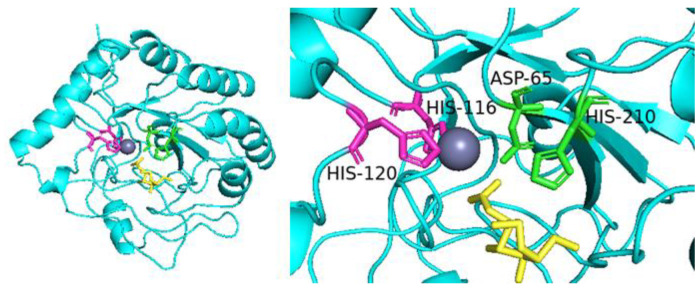
*Sb*CDA three-dimensional structure model and molecular docking results.

**Figure 7:**
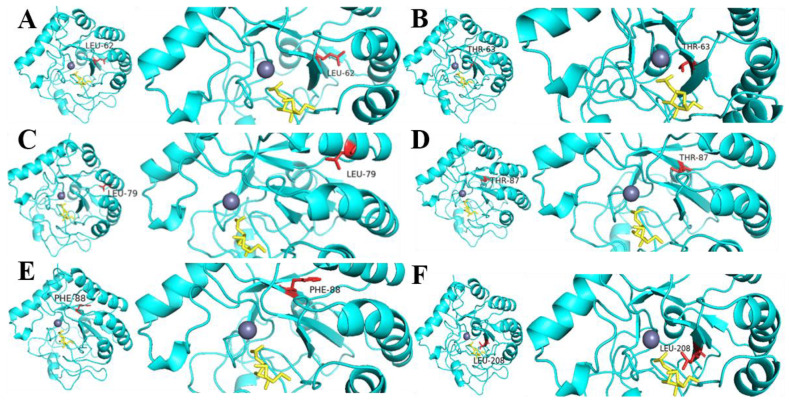
Molecular structure of *Sb*CDA. (**A**) Leu62; (**B**) Thr63; (**C**) Leu79; (**D**) Thr87; (**E**) Phe88; (**F**) Leu208.

By sequence alignment, we selected 19 amino acids (the blue dots in Figure 1) in the conserved region and mutated them into alanine. The enzyme activity of these 19 mutants was studied (Figure 5). The mutants *Sb*CDA62, *Sb*CDA63, *Sb*CDA77, *Sb*CDA79, *Sb*CDA87, *Sb*CDA88, *Sb*CDA156, and *Sb*CDA208 showed relatively obvious changes in enzyme activity. Then, to explore the role of the aforementioned amino acids in the structure, we established a homology model of *Sb*CDA using eight crystal structures as templates (PDB ID: 5LFZ [39], 6H8L [40], 2C1G [41], 5NC6 [42], 7AX7 (https://www1.rcsb.org/structure/7AX7, accessed on 16 November 2022), 1NY1 (https://www1.rcsb.org/structure/1NY1, accessed on 16 November 2022), 5O6Y (https://www1.rcsb.org/structure/5O6Y, accessed on 16 November 2022), and 4L1G [43]). The homology model (Figure 6) indicated that the His116 residues and His120 residues can form coordination bonds with Zn^2+^ and that Asp65 and His210 are the general base residue and the general acid residue for catalysis, respectively [21]. The three-dimensional protein structure diagram (Figure 7) shows that Leu62, Thr63, Leu79, Thr87, Phe88, and Leu208 of *Sb*CDA are around the catalytic site of the metal triplet. Therefore, we selected *Sb*CDA62, *Sb*CDA63, *Sb*CDA79, *Sb*CDA87, *Sb*CDA88, and *Sb*CDA208 for saturation mutation to search for mutants with higher enzyme activity.

### 2.6. SbCDA Saturation Mutations at Six Amino Acid Sites

**Figure 8:**
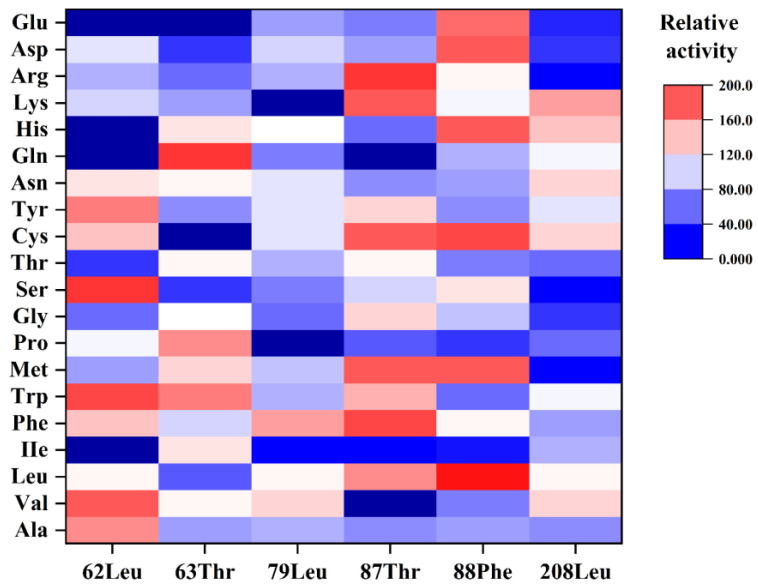
Relative enzyme activity of the mutants after saturation mutation, with the original enzyme activity as 100%. The colors represent the level of enzyme activity. Red means the enzyme activity is higher than the original protein. The redder the color, the higher the enzyme activity, and blue represents the opposite.

**Figure A3:**
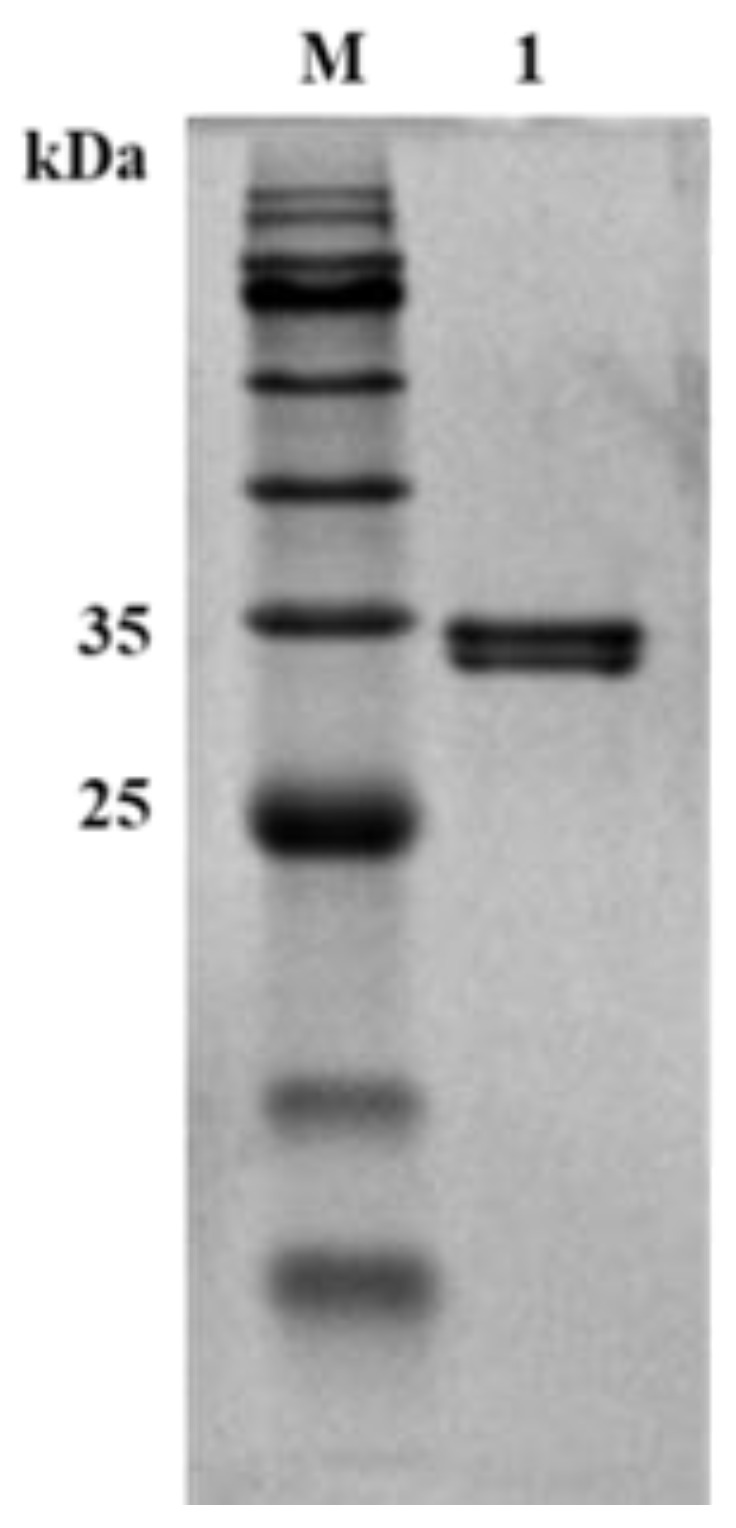
SDS-PAGE analysis of purified *Sb*CDA63G. Lane M: protein molecular mass marker; Lane 1: purified *Sb*CDA63G.

Based on the results of single-point mutation and structural analysis, saturation mutations were prepared on six amino acids at positions 62, 63, 79, 87, 88, and 208. The mutants were cultured, and the mutant proteins were collected under the same conditions. In order to comprehensively evaluate the enzyme activity and expression level, the activity of each mutant was determined by adding the same amount of supernatant (400 μL). As shown in Figure 8, mutant *Sb*CDA63G with Thr63 mutated to Gly, mutant *Sb*CDA79H with Leu79 mutated to His, and mutant *Sb*CDA87R with Thr87 mutated to Arg had relatively good enzyme activity. These three mutant strains were cultured, and the crude enzymes were collected. However, only mutant *Sb*CDA63G could be purified by Ni column chromatography. The protein concentrations of crude *Sb*CDA63G and purified *Sb*CDA63G were 1.9 mg/mL and 0.2 mg/mL, respectively. In addition, the specific enzyme activities of crude enzyme and purified *Sb*CDA were 35.9 U/mg and 115.9 U/mg, respectively. The purification factor was 3.2. SDS–PAGE analysis (Figure A3) showed that the obtained protein band was consistent with the predicted result. The mutant *Sb*CDA63G protein had a molecular weight of approximately 32 kDa.

### 2.7. Characterization of Mutant SbCDA63G

**Figure 9:**
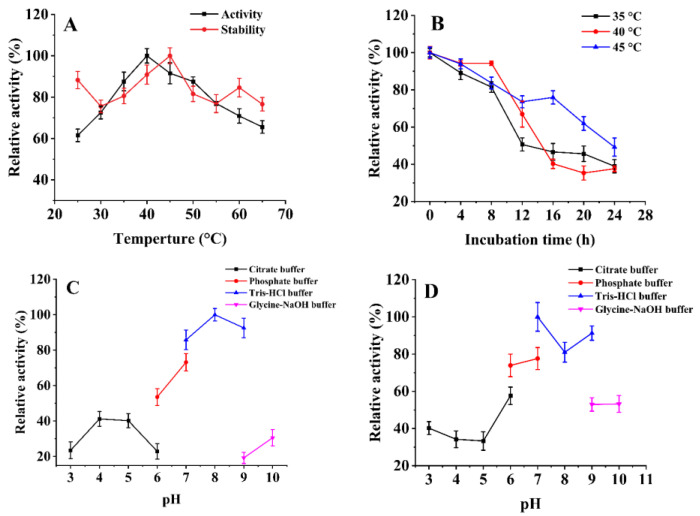
Biochemical characteristics of *Sb*CDA63G. (**A**) Effects of temperature on the activity of *Sb*CDA63G. Conditions for stability measurement: Tris-HCl buffer (10 mM, pH 7.0), incubation for 6 h. (**B**) Effects of temperature on the thermostability of *Sb*CDA63G. (**C**) Effects of pH on the activity of *Sb*CDA63G: citrate buffer (10 mM, pH 3.0–6.0), phosphate buffer (10 mM, pH 6.0–7.0), Tris-HCl buffer (10 mM, pH 7.0–9.0), and glycine-NaOH buffer (10 mM, pH 9.0–10.0). Reaction temperature: 40 °C. (**D**) Effects of pH on the stability in citrate buffer (10 mM, pH 3.0–6.0), phosphate buffer (10 mM, pH 6.0–7.0), Tris-HCl buffer (10 mM, pH 7.0–9.0), and glycine-NaOH buffer (10 mM, pH 9.0–10.0). Incubation conditions: 40 °C, incubation for 12 h. Using GlcNAc of 10 g/L as substrate throughout all measurements. All measurements were determined in triplicate, and the error bars indicate the standard deviations (*n* = 3).

When it comes to the optimum temperature, *Sb*CDA63G displayed its highest activity at 40 °C. When it comes to the stability of the enzyme at different temperature, *Sb*CDA63G showed more than 75% residual activity from 25 °C to 65 °C after being incubated for 6 h (Figure 9A). The half-lives of *Sb*CDA63G at 35, 40, and 45 °C were 12, 15, and 24 h, respectively (Figure 9B). The optimal pH of *Sb*CDA63G was 8.0. More than 75% of the activity of *Sb*CDA63G was retained after incubation for 12 h in a pH range from 7.0 to 9.0 (Figure 9D). *Sb*CDA63G has the greatest stability in a Tris-HCl buffer solution at pH 7.0.

## 3. Discussion

In this study, *Sb*CDA from *S. bacillaris* was obtained and investigated. The optimal temperature of *Sb*CDA was 35 °C under the reaction conditions of pH 7.0 Tris-HCl buffer (Figure 2A). In general, the optimum temperatures of CDAs from fungi (generally 50–60 °C) are higher than those of CDAs from bacteria [28,30,44,45,46,47,48,49]. Theoretically, the higher the temperature, the lower the stability of the enzyme, but in this study, the stability became higher at 45 °C (Figure 2A), probably because of the activation effect of temperature on the enzyme, but this phenomenon has not been found in other chitin deacetylases [30,33,34,35,36,39,40,41,42,50,51]. In addition, *Sb*CDA exhibited below 50% residual activity after incubation at 30, 35, and 40 °C for 24 h, indicating that the stability of *Sb*CDA is not very good. Therefore, one of the purposes of mutation is to improve the stability of *Sb*CDA. The optimum pH of 8.0 for *Sb*CDA is similar to those of CDAs derived from *Bacillus licheniformis*, *Nitratireductor aquimarinus* MCDA3-3, and *Vibrio cholerae* [45,52,53]. The high activity and good stability of *Sb*CDA in Tris-HCl buffer from pH 7.0–9.0 further support the idea that *Sb*CDA is an alkaline chitin deacetylase [54]. Activation by Mn^2+^ and inhibition by Na_2_EDTA indicate that *Sb*CDA is metal ion-dependent. In fact, the relationships between metal ions and the activity of almost all CDAs have been studied. For instance, Cd^2+^, Co^2+^, and EDTA ions inhibited the activity of *Me*CDA from *M. esteraromaticum* while K^+^, Li^+^, and Sr^2+^ ions strongly enhanced the enzyme’s activity [54]. ClCDA from *Colletotrichum lindemuthianum* was inhibited by Zn^2+^, Mn^2+^, and Cu^2+^, whereas it was not inhibited by Na^+^, K^+^, Li^+^, Mg^2+^, or Ca^2+^. Co^2+^ could improve the activity of ClCDA [55]. We cannot say for certain which metal ions have an inhibitory effect on the activity of CDAs and which metal ions could promote their activity, but it is worth making sure that most CDAs are metal ion-dependent and that EDTA can inhibit the activity of CDAs. In terms of substrate preference, *Sb*CDA and most reported CDA enzymes are mainly active on soluble chitins, such as colloidal chitin, and soluble chitosaccharides, but they have no activity against natural chitin [56]. However, MCDA02 from *Microbacterium esteraromaticum* and MCDA3-3 from *Nitratireductor aquimarinus* showed deacetylase activity toward α-chitin [54,56]. Most CDAs have no activity toward GlcNAc, while *Sb*CDA in our study has activity toward GlcNAc. If *Sb*CDA were used in combination with other enzymes that have different substrate preferences, different substrates could be utilized more efficiently, and a wide variety of chitosan oligosaccharides which have various physiological activities could be produced.

The enzymatic activity and stability of *Sb*CDA did not meet our expectations, and we modified CDA by single-point mutation and saturation mutation based on multiple sequence alignment to obtain a more efficient and stable CDA. The result of sequence alignment showed that there were 21 completely conserved amino acid sites among CDAs participating in the comparison (as shown by the red squares in Figure 1). According to previous research, CDAs operate by metal-assisted acid/base catalysis [57]. Asp65 and His210 (as shown by purple triangles in Figure 1) are the general base residue and the general acid residue for catalysis, respectively [21]. The other 19 completely conserved sites of CDAs are marked by blue dots. Previous studies have suggested conserved motifs that affect enzyme activity; however, this consensus can be improved [21]. Therefore, we speculated that our 19 highly conserved sites might influence the activity of *Sb*CDA, and we mutated these 19 amino acids (the blue dots in Figure 1) in the conserved region into alanine, which is a chiral amino acid with the shortest side chain [58]. Then, we studied the enzyme activity of these 19 mutants.

Among the 19 mutants, *Sb*CDA62, *Sb*CDA63, *Sb*CDA77, *Sb*CDA79, *Sb*CDA87, *Sb*CDA88, *Sb*CDA156, and *Sb*CDA208 showed the greatest alterations in enzyme activity, indicating that the change of amino acids at these positions is promising to improve the enzymatic activity of the enzyme. Meanwhile, the results of homology modeling illustrated that Leu62, Thr63, Leu79, Thr87, Phe88, and Leu208 were near the catalytic site of the metallic triad. Combining these two aspects, six amino acid sites (positions 62, 63, 79, 87, 88, and 208) underwent saturation mutagenesis. Among all mutants, *Sb*CDA63G, *Sb*CDA79H, and *Sb*CDA87R had relatively good enzyme activity. However, only *Sb*CDA63G could be purified. Therefore, the next inquiry of enzymatic properties was conducted using the mutant *Sb*CDA63G.

*Sb*CDA63G has higher activity and better stability compared with *Sb*CDA (Figure 9). The specific enzyme activity of *Sb*CDA63G was 115.9 U/mg, while that of *Sb*CDA was 49.6 U/mg, indicating that the activity of *Sb*CDA63G increased by approximately 133%. The increase in activity may be due to the increased flexibility of the main chain and less entropy loss during folding after mutation to Gly [59]. *Sb*CDA63G had better thermostability than *Sb*CDA, suggesting that the mutant is more suitable for industrial applications than *Sb*CDA [60]. A possible explanation for the increase in thermal stability is that the interaction between Gly and the nearby charged amino acids (D, E, K, and R) is more beneficial to the stability of the protein [61]. The mutation did not affect the optimal pH of the enzyme, which was 8.0 for both *Sb*CDA63G and *Sb*CDA (Figure 9C and Figure 2C). *Sb*CDA63G has its greatest stability in Tris-HCl buffer solution at pH 7.0, compared to pH 8.0 for *Sb*CDA.

Our study indicates that Thr63 has a great effect on enzyme activity. The mutation of Thr to Gly could improve the activity and stability of *Sb*CDA, making it more suitable for industrial production.

## 4. Materials and Methods

### 4.1. Materials

A One Step Cloning Kit and Taq polymerase were purchased from Vazyme (Nanjing, China). High-fidelity DNA polymerase was obtained from Toyobo (Osaka, Japan). K-ACET was purchased from Megazyme (Bray, Ireland). A Bacteria DNA Kit and a Plasmid Extraction Kit were purchased from Tiangen (Beijing, China). A Gel Extraction Kit was purchased from Omega (Guangzhou, China). Chitin was purchased from Sinopharm Group (Shanghai, China). Ni-NTA Superflow was obtained from Qiagen (Duesseldorf, Germany). N-acetyl glucosamine (GlcNAc), diacetylchitobiose ((GlcNAc)_2_), triacetylchitotriose ((GlcNAc)_3_), tetraacetylchitotetraose ((GlcNAc)_4_), acetylchitopentaose ((GlcNAc)_5_), and acetylchitohexaose ((GlcNAc)_6_) were purchased from Qingdao Bozhihuili Biotechnology (Qingdao, China). Other chemical reagents used in this study were of analytical grade unless specifically indicated. Colloidal chitin and carboxymethyl chitin were prepared according to previously reported methods [50,51].

### 4.2. Strains and Culture Conditions

*Streptomyces bacillaris* was activated in tryptic soy broth (TSB) medium containing 1.5% (*w*/*v*) tryptone, 0.5% (*w*/*v*) soya peptone, and 1% (*w*/*v*) NaCl. DH5α chemically competent cells were cultivated at 37 °C in Luria–Bertani (LB) medium composed of 0.5% (*w*/*v*) yeast extract, 1% (*w*/*v*) tryptone, and 1% (*w*/*v*) NaCl with 0.05 g/L kanamycin when needed. *Bacillus subtilis* WB800 was grown in LB medium at 37 °C. Thallus growth medium (GM) used for culturing *B. subtilis* WB800 contained 0.5% (*w*/*v*) yeast extract, 1% (*w*/*v*) tryptone, 1% (*w*/*v*) NaCl, and 9.1% (*w*/*v*) sorbitol. Electrotransfer medium (ETM) for the preparation of competent *B. subtilis* cells contained 9.1% (*w*/*v*) sorbitol, 9.1% (*w*/*v*) mannitol, and 10% (*v*/*v*) glycerol. Thallus recovery medium (RM) for the resuscitation of transformed cells contained 0.5% (*w*/*v*) yeast extract, 1% (*w*/*v*) tryptone, 1% (*w*/*v*) NaCl, 9.1% (*w*/*v*) sorbitol, and 6.9% (*w*/*v*) mannitol.

### 4.3. Bioinformatics Analysis, Homology Modeling, and Molecular Docking of CDAs

ExPASy ProtParam (https://web.expasy.org/protparam/, accessed on 16 November 2022) was used to calculate the isoelectric point and molecular weight. Both ESPript and ClustalX 2.1 were used to compare the amino acid sequences of chitin deacetylases (PesCDA (Genbank: A0A1L3THR9) [34], PdaA (Genbank: AFQ56715) [25], ClCDA (Genbank: Q6DWK3) [35], AnCDA (Genbank: XP_659456) [30], PgtCDA (Genbank: XP_003323413) [39], and AnCDA2 (Genbank: ACF22101.1)) with *Sb*CDA. The MODELLER software (version 9.23) was used to establish the homology model of the chitin deacetylase *Sb*CDA using eight crystal structures (PDB ID: 5LFZ [39], 6H8L [40], 2C1G [41], 5NC6 [42], 7AX7 (https://www1.rcsb.org/structure/7AX7, accessed on 16 November 2022), 1NY1 (https://www1.rcsb.org/structure/1NY1, accessed on 16 November 2022), 5O6Y (https://www1.rcsb.org/structure/5O6Y, accessed on 16 November 2022), and 4L1G [43]) as templates. The geometric conformation of the protein model was observed using PyMOL (Version 2.4.1). SAVES (http://services.mbi.ucla.edu/SAVES/, accessed on 16 November 2022), PROCHECK ERRAT, and VERIFY3D were used for further evaluation of the geometric conformation of the protein model. The molecular structure of GlcNAc was obtained from the ZINC database (http://zinc.docking.org/, accessed on 16 November 2022). The AutoDock (version 4.2.6) software (http://mgltools.scripps.edu/, accessed on 16 November 2022) was used for molecular docking. Finally, the docking site conforming to the catalytic conditions was selected as the initial conformation for analysis.

### 4.4. Construction of the SbCDA Expression Plasmid and Site-Directed Mutation

Genomic DNA was extracted from *S. bacillaris* using the Bacteria DNA Kit. Primer synthesis for the amplification of the *Sb*CDA gene, construction of mutant plasmids, and linearization of the p43NMK vector were conducted at Sangon Biotech Co., Ltd. (Shanghai, China). PCR products were purified and then ligated into the pP43NMK vector, which contains a 6×His tag, according to the instructions of the One Step Cloning Kit manufacturer. The recombinant and mutant plasmids were transformed into DH5α chemically competent cells for propagation of the plasmids. The Plasmid Extraction Kit was used to recover the plasmids. Then, the recombinant and mutant plasmids were electrotransformed into *B. subtilis* WB800 chemically competent cells to obtain transformants and mutant strains, respectively.

### 4.5. Expression and Purification of SbCDA and the Mutant Enzymes

To express the target enzymes, the transformants and mutant strains were grown in LB medium with shaking (220 rpm) for 20 h at 37 °C. After culture, the supernatant was obtained by centrifugation at 8000 rpm for 15 min at 4 °C to collect the crude enzyme solution. The activity of the crude enzyme solution was measured, and the mutant with an obvious enzyme activity change was selected for saturation mutation. Then, the mutant with the highest enzyme activity was selected to evaluate its properties. The enzyme was purified by a Ni-NTA Superflow column eluted with a gradient of imidazole (10, 50, 80, 100, 200, and 500 mM) in 50 mM Tris-HCl buffer (pH 8.0) and 500 mM NaCl. Finally, the purified protein was analyzed by SDS–PAGE. The content of protein was measured by Bradford assay [62]. The purified enzyme was used for further enzyme activity assays and biochemical characterization.

### 4.6. Chitin Deacetylase Activity Assay

Within all activity and stability tests, the enzyme activity assay was performed as previously described using the coupled enzymatic method with some modifications [36]. For the assay, 400 μL GlcNAc (10 g/L) was added to 400 μL Tris-HCl buffer solution (0.01 mol/L, pH 7.0), followed by 100 μL of enzyme solution, and the reaction system was incubated at 40 °C for 6 h. The activity of chitin deacetylase was characterized by measuring the content of acetic acid produced using the K-ACET acetic acid determination kit (Megazyme, Bray, Ireland).

One unit of enzymatic activity (U) was defined as the amount of enzyme (mg) required to produce 1 μmol of acetic acid per minute under the above conditions.

In order to verify the deacetylation products of chitin deacetylases, the hydrolysates were analyzed by mass spectrometry using an Agilent 6460 LC/MS.

### 4.7. Biochemical Characterization of SbCDA and the Mutant Enzymes

The impact of temperature on *Sb*CDA activity under standard conditions was studied from 25 to 65 °C, with a temperature interval of 5 °C. The same amount of enzyme solution was kept at the above temperature for 6 h to determine the stability. For the thermal stability assay, aliquots of enzyme were placed at 30, 35, and 40 °C for 0, 4, 8, 12, 16, 20, and 24 h. The residual enzyme activity was measured under standard conditions to determine the thermal stability. The enzyme half-life is calculated as the time it takes for the enzyme activity to drop to half of the initial enzyme activity.

Under standard conditions, the effect of pH buffers was examined in citric acid buffer (pH 3.0–6.0), phosphoric acid buffer (pH 6.0–7.0), Tris-HCl buffer (pH 7.0–9.0), and glycine sodium hydroxide buffer (pH 9.0–10.0). The concentration of all buffers was 10 mM. To investigate the effects of pH on the stability of *Sb*CDA, the enzyme was incubated in citric acid buffer (pH 3.0–6.0), phosphoric acid buffer (pH 6.0–7.0), Tris-HCl buffer (pH 7.0–9.0), and glycine sodium hydroxide buffer (pH 9.0–10.0) for 12 h, respectively. The residual activity was measured according to the standard method.

### 4.8. Effects of Metal Ions and Chemical Reagents on SbCDA Activity

To examine the effects of metal ions (Fe^3+^, Ca^2+^, Cu^2+^, Mg^2+^, Zn^2+^, Mn^2+^, Ni^2+^, Ba^2+^, Co^2+^, K^+^, and Na^+^) and a series of chemicals (disodium ethylenediaminetetraacetate (Na_2_EDTA) and sodium dodecyl sulfate (SDS)) on the activity of *Sb*CDA, they were added to the reaction mixture at final concentrations of 1 and 10 mM. The metal ions were each added to an enzyme solution, which was incubated for 1 h at 37 °C. Then, to measure the relative activity of *Sb*CDA, the reaction system was incubated at 40 °C for 6 h in Tris-HCl buffer (10 mM, pH = 7.0). The control group did not have metal ions or chemical reagents added under the same reaction system and reaction conditions as the experimental group.

### 4.9. Substrate Preference of SbCDA

Under standard conditions, chitin deacetylase was reacted with chitin, colloidal chitin, carboxymethyl chitin, GlcNAc, and (GlcNAc)_2_ to (GlcNAc)_6_ at a concentration of 10 mg/mL. The activity of chitin deacetylase on different substrates was determined.

## 5. Conclusions

In summary, a novel chitin deacetylase *Sb*CDA from *S. bacillaris* was expressed, purified, characterized, and molecularly modified. The phenomenon that Mn^2+^ can strongly improve the activity of *Sb*CDA needs to be further investigated to elucidate the activation mechanism of Mn^2+^. Different substrate preferences of *Sb*CDA from previously studied enzymes provide biological tools with potential for the production of multiple chitosan types. The activity of the mutant enzyme *Sb*CDA63G was 133% higher than that of *Sb*CDA. We speculate that the reason for this phenomenon is that Gly has low spatial bit resistance. When Thr was mutated to Gly at position 63, the entropy loss during folding decreased, and the flexibility of the main chain increased, which made it easier for the active center of the enzyme to deform and bind with the substrate. Moreover, the interaction between Gly and the nearby charged amino acids (D, E, K, and R) is more beneficial to the stability of the protein, and thus the optimal temperature and thermal stability of *Sb*CDA63G were also improved, making it more suitable for industrial production. Improved enzyme activity and stability would allow the mutant *Sb*CDA63G to achieve the same deacetylation as the original enzyme with less dosage. Our study demonstrates that mutation to Gly improves protein stability, providing a useful method for improving the properties of a chitin deacetylase and a potential tool for preparing chitosan or chitosan oligosaccharides from chitin.

## Data Availability

Not applicable.

## Sample Availability

Not available.

## Appendix A

The mass–charge ratio peaks of 162.1 and 180.1 in the mass spectrogram result are fragment ions of [GlcN+H]^+^ and [GlcN-H_2_O+H]^+^, respectively, which confirms the production of glucosamine (GlcN).

## Appendix B

The sequence of *Sb*CDA is as follows:

>*Sb*CDAMPKKMSVLGGGLAAALVATLTLALTGCSMETTAPASARRDAAPDAKGSFGRADCRKAKCIALTFDAGPGKDTAELLDILKEKKVSATFFLLGRNHVLKHPDTVRRIQDEGHEVANHTWTHKILTDEKPEEIRAELEKTQEAIEKITGKRPRLMRPPQGRTDDQVSGISKELGLSQVLWSATAKDYSTNDSALITQRILDQASRDGIILLHDIYKGTVPAVPGIIDALQKDGYTFVTVPELMAPAVPEPGTIYRP
